# Characteristics of Human and Mouse Orthologous Protein-Coding Nucleotide Sequences with Large G+C Content Variations

**DOI:** 10.2174/138920207783591708

**Published:** 2007-11

**Authors:** Hiroshi Nakashima

**Affiliations:** Department of Clinical Laboratory Science, Graduate Course of Medical Science and Technology, Division of Health Science, Kanazawa University, 5-11-80 Kodatsuno, Kanazawa 920-0942, Japan

**Keywords:** Human mouse orthologs, G+C content variation, nucleotide substitution, gene location, gene rearrangement.

## Abstract

Characteristics of human and mouse orthologous gene sequences which have large G+C content variations were investigated in this study. The orthologous gene pairs were classified into two groups according to the deviation between human and mouse G+C content at the third codon position (GC3) and were subsequently analyzed. In one group, mouse genes had higher GC3 than the corresponding human genes and in another group, human genes had higher GC3 than mouse. Furthermore, the orthologous pairs were separated based on the deviation between human or mouse GC3 and the G+C content at the third codon position of identical codons (IC3), to examine the effect of increased or decreased G+C content in human or mouse sequences. The nucleotide substitution patterns between human and mouse sequences in the two groups were remarkably distinct, and consistent with the state of G+C-rich or G+C-poor sequences. The effect of increase or decrease of G+C content in human or mouse sequences was not clear in the nucleotide substitution patterns. The chromosomal locations of human and mouse orthologous gene pairs were different between the two groups. The genes located on an identical syntenic segment showed the trend of having similar G+C content. Moreover, the same gene order of some genes on different chromosomes of both species demonstrated the gene rearrangements between human and mouse. Our study indicated that the chromosomal locations and rearrangements are associated with the GC3 variation between human and mouse sequences.

## INTRODUCTION

Mammalian genomes consist of long DNA stretches (over hundreds of kilobases) with varying G+C content known as isochores [1]. Some G+C-poor isochores have G+C contents as low as 35%, while G+C-rich isochores have G+C contents as high as 60%. The isochore structure of the genome is reported by many investigators [2-8]. Studies have indicated that several homologous mammalian genes occupying different chromosomal positions differ considerably in their base composition and codon usage [1,2,9]. Human α - and β -globin genes are an example of this position-dependent variation. The α -globin gene cluster occupies a G+C-rich region, while the β -globin gene resides in a G+C-poor region [1]. Genes with a high G+C content at the third codon position, or high GC3 (e.g. >80% G+C), are almost always surrounded by long G+C-rich (e.g. 55-65% G+C) genomic sequences, while those with a low GC3 (e.g. <50% G+C) are surrounded by long A+T-rich sequences (e.g. 35-45% G+C) [10,11]. Many studies of the G+C content variations among mammals have been reported [12-16].

Recently, we reported a negative correlation of G+C content at silent substitution sites between orthologous human and mouse protein-coding sequences [17]. Several gene pairs examined showed significant differences in the G+C content at silent substitution sites: for example, human thymine-DNA glycosylase was A+T-rich at silent substitution sites, while the mouse sequence was G+C-rich at the corresponding sites. In contrast, human matrix metalloproteinase 23B was G+C-rich at silent substitution sites while the mouse sequence was A+T-rich at the corresponding sites. The G+C variation at the third codon position between human and mouse thymine-DNA glycosylase sequences was 22.6% and the G+C variation in matrix metalloproteinase 23B was 28.5%. The G+C variation at silent substitution sites reflected the G+C variation at the third codon position.

Of interest is to better understand the source of observed variations in G+C content. In this study, the characteristics of human and mouse orthologous sequences were investigated based on classifications in terms of G+C content deviation. The nucleotide substitution pattern between human and mouse sequences, where the G+C content in the mouse sequence is higher than that in the human sequence, is considered distinct from when the observed G+C content in the human sequence is higher than that in the mouse. Therefore, the orthologous gene pairs were analyzed separately according to the deviations between human and mouse GC3, and the effect of increased or decreased G+C content on the nucleotide substitution patterns was investigated. Furthermore, we assumed that the difference in G+C content between human and mouse sequences would be caused by significant increase or decrease in G+C content in either human or mouse sequences after divergence from their common ancestor. Assuming that the G+C content of identical codons corresponds to that of the common ancestor [18,19], human or mouse sequences, which show a larger G+C content deviation from the IC3, were selected for the analysis of nucleotide substitutions.

Classified genes were characterized and examined based on the comparison by their nucleotide substitution frequency between orthologous human and mouse sequences, amino acid substitution frequency, chromosomal location, and TA and GC skews.

## MATERIALS AND METHODS

### Orthologous Gene Pairs 

The human and mouse cDNA sequences were obtained from Reference Sequence Release 11 from the National Center for Biotechnology Information (NCBI) (ftp://ftp.ncbi.nih.gov/refseq/), and the protein-encoding nucleotide sequences were selected according to the feature table for the data. The amino acid sequences of 28,893 human cDNAs and 25,298 mouse cDNAs were obtained by translation, and the 3,776 pairwise alignments of human and mouse orthologous nucleotide sequences were prepared as previously described [17]. Alignments longer than 300 nucleotides without gaps were employed for the calculation of G+C content and nucleotide substitution frequency. The average identities of nucleotide sequences and amino acid sequences from the 3,776 alignments were 86% and 89%, respectively. 

The TA and GC skews of aligned sequences were calculated as follows: 

TA skew = (T-A)/(T+A)

GC skew = (G-C)/(G+C)

The TA and GC skews at the third codon positions were calculated similarly using the nucleotide compositions at the third codon position, and the skews were given as a percentage.

### Classification of Orthologous Sequences

Human and mouse orthologous sequence pairs were classified according to the deviation between human and mouse GC3. When a mouse sequence contained 10% higher GC3 than the corresponding human sequence, the pair was selected for the dataset as group 1. Similarly, when a human sequence contained 10% higher GC3 than the corresponding mouse sequence, the pair was chosen for the dataset as group 2. There were 703 orthologous pairs in group 1 and 472 pairs in group 2. The orthologous pairs were then further divided according to the absolute difference between IC3 and human or mouse GC3. When mouse GC3 showed stronger deviation from the IC3 than human GC3, the pair was categorized further into group 1.1 or group 2.1. Conversely, when human GC3 showed stronger deviation from the IC3 than mouse GC3, the pair was categorized further into group 1.2 or group 2.2. This subcategorization led to 198 pairs in group 1.1, 131 pairs in group 1.2, 468 pairs in group 2.1 and no pairs in group 2.2, when 5% deviation was used as a criterion of the classification. The pairs with no strong deviation were not categorized. The procedure of classification of orthologous pairs is shown in Fig. (**1**).

### Chromosomal Location

Chromosomal locations of genes were obtained from the annotation of Reference Sequence data from the NCBI. Gene location on a chromosome was obtained from the web site Map Viewer of the NCBI ( http://www.ncbi.nlm.nih.gov/mapview/).

## RESULTS

### Average G+C Content at the Third Codon Position and TA and GC Skews

The calculated averages of GC3 and IC3 for each group as well as the complete dataset are shown in Table **1a**. Analysis of human GC3 revealed a great difference of 40% between group 1 and 2, while the corresponding difference in mouse was 10.8% indicating that human sequences have a wider range of GC3 than mouse. The averages of mouse GC3 in group 1.1 and 1.2 deviated approximately 20%, similar to that observed with human GC3. Due to the definition of classification, for example, there are two possible cases in group 1.1; (1) mouse GC3 is higher than IC3 and human GC3 is lower than IC3. (2) both mouse GC3 and human GC3 are higher than IC3. The occurrence of two possible cases is listed in Table **1b**. Both in groups 1.1 and 1.2, most of orthologous pairs showed that mouse GC3 is higher than IC3 and human GC3 is lower than IC3. In group 2.1, most of orthologous pairs showed that both human GC3 and mouse GC3 are lower than IC3. The histogram of IC3 indicated that the orthologous pairs in group 1.1 and 1.2 are almost separated in terms of IC3 (data not shown). The average of IC3 in group 1.1 was 37.0%, which was closer to that of human GC3 than that of mouse. This indicated that GC3 had deviated mainly in mouse sequences by increasing after the two species separated from their common ancestor. On the other hand, the average of IC3 in group 1.2 was 65.0%, which was closer to that of mouse GC3 than in humans, indicating that human sequences experienced major change in GC3 by decreasing GC3 in these cases. The average of IC3 in group 2.1 was 83.8%, close to that of human GC3 and suggesting that the mouse GC3 had decreased. Virtually, all data in group 2 were included in group 2.1, and no data was observed in group 2.2. The averages of GC3 and IC3 vary significantly among groups 1.1, 1.2 and 2.1. Table **1a** indicated that human GC3-poor orthologs are less in GC3 content than their mouse counterparts (group 1.1), and human GC3-rich orthologs are richer in GC3 content than their corresponding mouse sequences (group 2.1). The averages of human and mouse GC3 in the entire dataset were close, therefore, the characteristic features of GC3 were specific when comparing between the classified orthologous pairs.

The deviation of both TA and GC skews between human and mouse sequences at the third codon position was greater than those from a whole sequence, and are indicated in Table **1a**. Averages of human and mouse TA and GC skews were different among the groups, with a general trend of TA skews increasing with the increase of GC3, and GC skews increasing with the decrease of GC3. The averages of both human and mouse GC skew in group 2.1 were negative, which indicated that cytosine is more favorable than guanine at the third codon position in a sequence of high GC3 content. 

### Nucleotide Substitution Frequency 

The frequency of nucleotide substitutions in the orthologous gene pairs of group 1.1 is shown in Table **2a**. Nucleotide substitutions were observed in 14% (41,412/289,206) of nucleotides within the 198 orthologous pairs. Substitutions between A in human and G in mouse (25.7%) and T in human and C in mouse (25.4%) were dominant. The substitution frequencies between A/T in human and G/C in mouse, and G/C in human and A/T in mouse were 64.5% and 22.5%, respectively. This is consistent with the characteristic of classified orthologous pairs, i.e., GC3 in mouse is higher than that in human. When only the substitutions at synonymous sites were considered, the substitution frequencies between A/T in human and G/C in mouse, and G/C in human and A/T in mouse were 69.6% and 20.3%, respectively. The richness of G+C content in mouse sequences is greater at synonymous site substitutions; substitutions between T in human and C in mouse (30.7%), and A in human and G in mouse (27.7%) were enlarged at synonymous site substitutions. Ten frequently observed amino acid substitutions were also listed in Table **2a**, stating that amino acid replacement between Ile in human and Val in mouse showed the highest substitution frequency, and three reverse replacements such as Ile-Val and Val-Ile, Thr-Ala and Ala-Thr, Glu-Asp and Asp-Glu were also observed.

The nucleotide substitution frequency of orthologous pairs in group 1.2 was similar to that of group 1.1 (data not shown). Although we predicted that the substitution frequency patterns would differ for the two groups, no clear difference was observed in the substitution patterns between the two groups. Moreover, the ten frequently observed amino acid replacements in group 1.2 were identical with those in group 1.1 (Table **2a**). These results indicated that increase or decrease of G+C content in human or mouse sequences does not yield different nucleotide substitution patterns.

The frequency of nucleotide substitutions of orthologous pairs in group 2.1 is presented in Table **2b**, and nucleotide substitutions were observed in 16% (92,334/1,583,867) of nucleotides within the 468 orthologous pairs. In group 2.1, substitutions between C in human and T in mouse (25.2%), and G in human and A in mouse (21.1%) were dominant, while the substitution frequencies between A/T in human and G/C in mouse, and G/C in human and A/T in mouse were 22.7% and 59.6%, respectively. These trends are consistent with the characteristics of selected orthologous pairs, i.e., GC3 in human is higher than that in mouse. When substitutions only at synonymous sites were considered, the substitution frequencies of A/T in human and G/C in mouse, and G/C in human and A/T in mouse were 19.7% and 64.7%, respectively. G+C content preference in human sequence is greater at synonymous site substitutions. Substitution of C in human and T in mouse (31.8%) was greater at synonymous site substitutions, though substitution of G in human and A in mouse (21.2%) was similar. The ten frequently observed amino acid substitutions were again determined, and listed in Table **2b**, with the most frequently observed amino acid replacement being Ala in human and Thr in mouse. In the top ten amino acid substitutions, only one reverse replacement of Ala-Thr and Thr-Ala was observed. Frequently observed amino acid substitutions in group 1.1 (e.g. Ile in human vs. Val in mouse) and in group 2.1 (e.g. Ala in human vs. Val in mouse) were different. The codons of Ile, Val and Ala are AT-rich, neutral and GC-rich, respectively, therefore, difference of amino acid substitutions could be attributed to the opposite GC3 content in the two groups. 

### Chromosomal Locations of Orthologous Genes

Chromosomal locations of 3,776 orthologous human and mouse gene pairs were examined, the available 3,769 locations are shown in Table **3**. Human genes corresponding mouse genes on mouse chromosome 1 were observed on human chromosomes 1, 2, 3, 5, 6, 8, 13, and 18. On the other hand, mouse genes corresponding human genes on human chromosome 1 were observed on mouse chromosomes 1, 2, 3, 4, 5, 6, 8, 11, and 13. This scattered location of orthologous gene pairs suggested a large number of gene rearrangements which is consistent with the study of genome sequence comparison [20-23]. However, human genes on human chromosome 20 were only corresponding to mouse genes on mouse chromosome 2. Human genes on human chromosome 17 were almost corresponding to mouse genes on mouse chromosome 11, and human genes on human chromosome 14 were corresponding to mouse genes on mouse chromosomes 12 and 14. This result indicated that human genes on some chromosomes do not scattered as compared to mouse genes. Human genes on human X chromosome were almost corresponding to mouse genes on mouse X chromosome. Table **3** also indicates the number of gene pairs of groups 1 and 2. In the top three gene pairs of group 1, thirty one pairs were observed on human chromosome 4 and on mouse chromosome 5, thirty pairs on human chromosome 1 and on mouse chromosome 1, as well as on human chromosome 12 and on mouse chromosome 10. For the top three gene pairs of group 2, thirty nine pairs were observed on human chromosome 19 and on mouse chromosome 7, twenty nine pairs on human chromosome 16 and on mouse chromosome 17, and twenty five pairs on human chromosome 17 and on mouse chromosome 11, as well as on human chromosome 11 and on mouse chromosome 19. This result indicated that chromosomal locations of orthologous pairs were different between groups 1 and 2. Orthologous genes from different groups reside on distinct chromosomal positions, and the orthologous genes within the same group frequently appeared in series along a chromosome. As an example, five orthologous human genes in groups 1.1, 1.2 and 2.1 and their corresponding mouse genes are listed in Table **4**. The gene order on a chromosome of both species was conserved, indicating gene rearrangements between human and mouse. Five orthologous human and mouse genes in groups 1.1, 1.2 and 2.1 reside in a span of approximate 7, 3 and 1Mbp along the chromosome, respectively. The gene density was high in a GC3-rich region (group 2.1) consistent with the report of Bernardi *et al*. [1]. The GC3 content in each group was in a narrow range, which indicated that the genes in identical syntenic segments have similar GC3 content. This is consistent with the previous reports [10,11]. However, we did observed several cases that a gene with differing GC3 content appears in the middle of cluster of genes of similar GC3 content. 

The TA or GC skew value of genes at the third codon position showing deviation between human and mouse sequences are shown in Table **4**. The skew values differ considerably between orthologous human and mouse genes, indicating an asymmetry in the substitution patterns in the two DNA sequences. The GC skews of human genes in group 1.1 were larger than those of mouse counterparts, while the TA skews of human genes in group 1.2 were almost larger than those of mouse counterparts, and the TA skews of mouse genes in group 2.1 were conversely larger than human counterparts. The existence of compositional asymmetries has been reported to be associated with replication origins. TA and GC skews display significant changes or V-shaped profiles at the replication origins in bacteria [24,25] and mammalian genomes [26], thus the relative positional changes against the replication origins yielded by rearrangements might be the cause of the significant difference of skew values. Experimentally determined appropriate replication original sites on human and mouse chromosomes have yet to be established to test the above hypothesis. 

## DISCUSSION

**Fig. (1):**
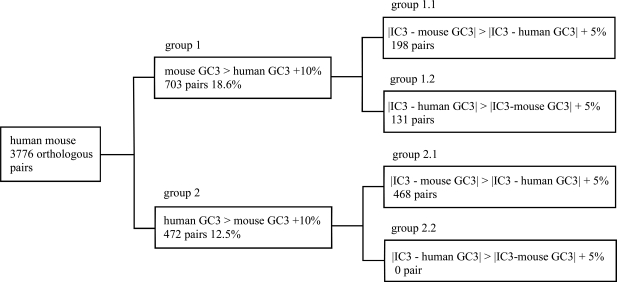
Schematic of the classification of orthologous human and mouse sequences.

Several human genes are G+C-rich at the third codon position and their corresponding mouse genes are G+C-poor, while other human genes are G+C-poor with their corresponding mouse gene are G+C-rich. To analyze the characteristics of orthologous human and mouse sequences with significant GC3 variation, classification of orthologous sequences is essential. In our study, we used 10% as a threshold; if the threshold was lowered to 5%, the number of pairs in groups 1 and 2 were 1373 (36.4%) and 1045 (27.7%), respectively. When these two groups were further separated according to the deviation from the IC3 (same methodology as presented in Fig. **1**), the number of orthologous pairs in groups 1.1, 1.2, 2.1, and 2.2 were 343, 284, 982, and 2, respectively. Membrane associated DNA-binding protein and TCDD-inducible poly (ADP-ribose) polymerase were two of the gene products in group 2.2. The averages of human GC3, mouse GC3 and IC3 in group 2.2 were 43.0, 36.3 and 36.5%, respectively. The averages of GC3 and IC3 of other groups using the 5% threshold showed similar trends to those presented in Table **1a**. However, the deviation between human GC3 and mouse GC3 were smaller in this case compared to the data presented in Table **1b**. The nucleotide substitution frequency of genes in group 1.1 and 1.2 were largely similar to that of Table **2a**, however, the deviation between A/T and G/C frequency was smaller compared to Table **2a**. The ten frequently observed amino acid substitutions in groups 1.1 and 1.2 were virtually identical as in Table **2a**. Therefore, we concluded that the results would not differ significantly if 5% was used as a threshold in classification.

The maximum and minimum GC3 content in the entire human sequences were 97.7 and 24.0%, and those of mouse were 97.9 and 29.1%, respectively, indicating that the GC3 content spanned an extremely wide range in both human and mouse sequences. In this study, human and mouse sequences were classified in terms of GC3 content deviation and subsequently analyzed. The GC3 averages of group 1.1 and 1.2 for both human and mouse sequences deviated approximately 20%, and the GC3 content of both human and mouse sequences in groups 1.1 and 1.2 clustered around their averages within 10 %. Therefore, human and mouse sequences in groups 1.1 and 1.2 were almost separated in term of GC3 content. In other words, both human and mouse sequences in group 1.1 and 1.2 were rather homogeneous in terms of GC3 content. This trend holds for both the human and mouse sequences in group 2.1. 

Chromosomal locations of human and mouse genes in groups 1 and 2 were biased. Genes in the same group were often observed as a cluster on chromosomes, and they showed similar GC3 content. Conserved gene order of both species indicated the rearrangements of genes. Therefore, it is considered that chromosomal locations and rearrangements are related with the large deviation of GC3 content between orthologous human and mouse sequences. Chromosomal location of genes would change by rearrangement suggesting that rearrangements might be a major factor that generates GC3 content variation. Establishment or understanding of rearrangement events between human and mouse chromosomes could provide further clues to the cause of GC3 variation.

## Figures and Tables

**Table 1a.** Average of G+C Content (#x0025;) and AT/GC Skews (%) at the Third Codon Positions

Group	No. of Sequences	Human	Common	Mouse	Human	Mouse	Human	Mouse
		GC3	IC3	GC3	TA skew	TA skew	GC skew	GC skew
1	703	41.0	48.1	56.0	5.2	5.7	8.4	3.6
1.1	198	33.6	37.0	48.4	3.3	4.5	12.2	6.5
1.2	131	52.3	65.0	67.5	8.0	7.7	3.4	0.7
2	472	81.0	83.6	66.8	14.9	13.3	-4.6	-3.1
2.1	468	81.2	83.8	66.9	14.9	13.3	-4.6	-3.1
whole data	3776	57.9	61.9	59.2	9.5	9.8	1.6	0.4

GC3 stands for G+C content at the third codon position.

IC3 stands for G+C content at the third codon position of identical codons.

**Table 1b.** Relation between GC3 and IC3

Group	Relation between GC3 and IC3	Occurrence
1.1	mouse GC3 > IC3 > human GC3	98% (194/198)
	mouse GC3 > IC3, human GC3 > IC3	2% (4/198)
1.2	mouse GC3 > IC3 > human GC3	85% (112/131)
	IC3 > mouse GC3, IC3 > human GC3	15% (19/131)
2.1	IC3 > human GC3, IC3 > mouse GC3	85% (398/468)
	human GC3 > IC3 > mouse GC3	15% (70/468)

**Table:** 

Human	A	Mouse T	G	C
A	* * *	3.8	25.7	8.0
T	3.4	* * *	5.4	25.4
G	9.6	1.7	* * *	2.9
C	2.6	8.6	2.9	* * *

**Table:** 

Human		Mouse	Whole	Synonymous
A/T	<->	G/C	64.5	69.6
G/C	<->	A/T	22.5	20.3
A/T	<->	T/A	7.2	6.8
G/C	<->	C/G	5.8	3.3

**Table:** Ten frequently observed amino acid substitutions.

No.	1	2	3	4	5	6	7	8	9	10
Human	I	K	N	T	E	D	V	S	A	V
Mouse	V	R	S	A	D	E	I	A	T	A

**Table:** 

Human	A	Mouse T	G	C
A	***	2.5	8.3	3.3
T	2.5	***	2.1	9.0
G	21.1	5.4	***	6.5
C	7.9	25.2	6.2	***

**Table:** 

Human		Mouse	Whole	Synonymous
A/T	<->	G/C	22.7	19.7
G/C	<->	A/T	59.6	64.7
A/T	<->	T/A	5.0	4.5
G/C	<->	C/G	12.7	11.1

**Table:** Ten frequently observed amino acid substitutions.

No.	1	2	3	4	5	6	7	8	9	10
Human	A	A	V	A	T	R	R	G	E	P
Mouse	T	V	I	S	A	Q	K	S	D	S

**Table 3.** Chromosomal Locations of Human and Mouse Orthologous Gene Pairs

			Human																						
Mouse	1	2	3	4	5	6	7	8	9	10	11	12	13	14	15	16	17	18	19	20	21	22	X	Y	Sum
1	107	95	1	0	1	9	0	11	0	0	0	0	3	0	0	0	0	5	0	0	0	0	0	0	232
	30, 0	13, 10	1, 0		1, 0	6, 0		1, 1										0, 1							52, 12
2	1	41	0	0	0	0	1	0	57	23	38	0	0	0	36	0	0	1	0	113	0	0	0	0	311
		17, 1							1, 16	5, 1	2, 2				6, 2					8, 21					39, 43
3	100	0	21	35	0	0	0	6	0	0	0	0	4	0	0	0	0	0	0	0	0	0	0	0	166
	22, 4		7, 0	17, 1				1, 0					4, 0												51, 5
4	161	0	0	0	0	7	0	9	61	0	0	0	0	0	0	0	0	0	0	0	0	0	1	0	239
	18, 19					2, 0		2, 0	8, 3																30, 22
5	5	18	0	93	1	0	77	0	0	0	0	57	14	0	0	0	0	1	0	0	0	4	1	1	272
	1, 0	0, 1		31, 12	1, 0		14, 14					18, 3	7, 1									2, 1			74, 32
6	2	27	45	4	0	0	57	0	0	3	0	44	0	0	1	0	1	0	0	0	0	2	0	0	186
		5, 1	1, 3	1, 0			8, 3			1, 0		19, 0										1, 0			36, 7
7	0	0	0	0	0	1	0	1	0	19	63	0	0	0	34	34	0	0	92	0	0	0	0	0	244
										3, 1	6, 10				3, 3	1, 8			3, 39						16, 61
8	11	0	0	24	0	1	0	28	1	1	0	0	11	0	0	87	0	0	40	0	0	0	0	0	204
	10, 0			6, 0				3, 3	1, 0	1, 0			5, 0			8, 11			2, 23						36, 37
9	0	0	87	0	0	18	5	0	0	1	67	0	0	0	61	0	0	0	13	0	0	0	0	0	252
			16, 1			8, 0	3, 1				12, 3				14, 5				0, 4						53, 14
10	0	3	0	0	0	52	0	0	0	20	0	71	0	0	0	0	0	0	24	0	11	7	0	0	188
		1, 1				19, 1				10, 1		30, 1							0, 18		0, 2	0, 1			60, 25
11	2	16	0	0	26	0	6	0	0	1	0	0	0	0	0	3	227	0	0	0	0	8	0	0	289
	0, 2	1, 1			6, 3											0, 2	16, 25					0, 1			23, 34
12	0	24	0	0	0	0	12	0	0	0	0	0	0	97	0	0	0	0	0	0	0	0	0	0	133
		5, 1					3, 0							23, 9											31, 10
13	5	0	0	1	56	25	6	1	16	5	0	0	0	0	0	0	0	0	1	0	0	0	0	0	116
	2, 0			1, 0	17, 7	3, 0	1, 0		7, 0	0, 1															31, 8
14	0	0	19	0	0	0	0	20	0	22	0	0	39	33	0	0	0	0	0	0	0	0	0	0	133
			4, 1					0, 2		3, 8			14, 0	4, 2											25, 13
15	0	0	0	0	27	0	0	56	0	0	0	36	0	0	0	0	0	0	0	0	0	49	0	0	168
					9, 1			6, 18				15, 0										6, 12			36, 31
16	0	2	60	0	0	0	0	1	0	1	0	2	0	0	0	25	0	0	0	0	28	14	1	0	134
		0, 1	19, 1							1, 0						2, 7					15, 0	0, 12			37, 21
17	0	24	2	0	4	75	0	0	0	0	0	0	0	0	0	35	0	5	18	0	4	0	0	0	167
		12, 0			2, 0	10, 2										0, 29		5, 0	0, 11		0, 2				29, 44
18	0	2	0	0	37	0	0	0	0	8	0	0	0	0	0	0	0	36	0	0	0	0	0	0	83
					10, 3					1, 0								12, 1							23, 4
19	0	1	0	0	0	0	0	0	15	50	73	0	0	0	0	0	0	0	0	0	0	0	0	0	139
									6, 1	13, 0	0, 25														19, 26
X	0	0	0	1	0	0	0	0	0	0	0	0	0	0	0	0	0	0	0	0	0	0	112	0	113
				1, 0																			1, 21		2, 21
Y	0	0	0	0	0	0	0	0	0	0	0	0	0	0	0	0	0	0	0	0	0	0	0	0	0
																									0, 0
sum	394	253	235	158	152	188	164	133	150	154	241	210	71	130	132	184	228	48	188	113	43	84	115	1	3,769
	83, 25	54, 17	48, 6	57, 13	46, 14	48, 3	29, 18	13, 24	23, 20	38, 12	20, 40	82, 4	30, 1	27, 11	23, 10	11, 57	16, 25	17, 2	5, 95	8, 21	15, 4	9, 27	1, 21	0, 0	703, 470

top column: the number of human and mouse orthologous gene pairs

bottom left column: the number of orthologous gene pairs of group 1.

mouse GC3 > human GC3 + 10%

bottom right column: the number of orthologous gene pairs of group 2.

human GC3 > mouse GC3 + 10%

**Table 4.** Human and Mouse Orthologous Gene Pairs

Gene Product	Human				Common	Mouse			
	Gene Location (Mbp)	Code	Skew	GC3	IC3	GC3	Skew	Code	Gene Location (Mbp)
** Group 1.1**	** chr 12p12**		**GC**				**GC**		**chr 6**
activating transcription factor 7 interacting protein	15	NM_018179	11.3	31.0	35.9	45.3	3.9	NM_019426	137
serine/threonine kinase receptor associated protein	16	NM_007178	15.7	31.6	35.7	46.1	8.7	NM_011499	138
phosphodiesterase 3A, cGMP-inhibited	21	NM_000921	-3.3	38.2	44.3	52.5	-18.2	NM_018779	141
solute carrier organic anion transporter family, member 1b	21	NM_019844	-1.7	32.5	29.6	41.2	-10.2	NM_020495	142
cytidine monophospho-N-acetylneuraminic acid synthetase	22	NM_018686	9.9	35.4	40.7	53.4	0.9	NM_009908	143
** Group 1.2**	**chr 12q13**		**TA**				**TA**		**chr 15**
NYD-SP28 protein	48	NM_033124	-4.8	59.8	74.1	72.3	-3.9	NM_153518	99
testis enhanced gene transcript (BAX inhibitor 1)	48	NM_003217	33.3	52.6	63.3	68.9	21.1	NM_026669	99
Rac GTPase activating protein 1	49	NM_013277	12.5	48.2	57.1	59.6	4.7	NM_012025	99
LETM1 domain containing 1	50	NM_015416	22.7	54.7	64.5	65.8	12.2	NM_134093	100
sodium channel, voltage gated, type VIII	51	NM_014191	0.4	58.4	69.5	71.5	-6.5	NM_011323	101
** Group 2.1 **	**chr 8q24.3**		**TA**				**TA**		**chr 15**
zinc finger CCCH type domain containing 3	145	NM_015117	9.8	81.5	88.8	66.7	28.3	NM_172121	76
epiplakin 1	145	NM_031308	-4.1	83.2	87.0	66.9	6.9	NM_144848	76
brain protein 16	145	NM_016458	6.9	81.7	84.7	69.7	18.8	NM_021555	76
5-oxoprolinase	145	NM_017570	22.7	81.5	82.6	61.9	21.4	NM_153122	76
solute carrier family 39 (zinc transporter)	146	NM_130849	-36.5	88.9	96.9	68.2	-4.8	NM_028064	76

chr stands for chromosome.

## ABBREVIATIONS

GC3: = G+C content at the third codon position
IC3: = G+C content at the third codon position of identical codons
